# Structure and Solvent Trapping in Polymer Brushes Under Poor-Solvent Conditions

**DOI:** 10.3390/ma19204320

**Published:** 2026-10-14

**Authors:** Piotr Polanowski, Andrzej Sikorski

**Affiliations:** 1 Department of Molecular Physics, Lodz University of Technology, Zeromskiego 114, 90-543 Lodz, Poland; ppolanow@p.lodz.pl; 2 Faculty of Chemistry, University of Warsaw, Pasteura 1, 02-093 Warsaw, Poland

**Keywords:** lattice model, Monte Carlo method, polymer brush, polymer conformation, solvent quality

## Abstract

**Highlights:**

- Solvent entrapment in polymer brushes at poor-solvent conditions.
- Monotonic brush collapse in a poor solvent is not generally valid.
- The solvent confinement affects brush collapse.

**Abstract:**

Polymer brushes exhibit complex structural responses to changes in grafting density and solvent quality, particularly under poor-solvent conditions. Although brush collapse has been extensively studied, the role of an explicit solvent in determining the structure and conformational response of densely grafted polymer layers remains insufficiently understood. In this work, we investigate the structure of polymer brushes composed of flexible linear homopolymer chains grafted onto an impermeable surface in an explicit solvent. A coarse-grained lattice model was studied using Monte Carlo simulations with the Cooperative Motion Algorithm, allowing systematic variation in chain length, grafting density, and solvent quality. Chain conformations, brush height, density profiles, and solvent distribution within the polymer layer were analyzed. The simulations show that brush response to decreasing solvent quality is not universally monotonic. Depending on grafting density and chain length, the radius of gyration and its component perpendicular to the grafting surface exhibit non-monotonic changes, while the brush develops heterogeneous polymer and solvent distributions under poor-solvent conditions. In particular, solvent molecules can become locally trapped within the polymer layer, producing polymer-rich and solvent-rich regions. These structural changes arise from competition between polymer–polymer interactions promoting chain collapse and solvent mixing entropy with solvent confinement. These results demonstrate that explicit solvent redistribution can play an active role in the structural organization of polymer brushes and that solvent trapping should be considered when describing their behavior under poor-solvent conditions. The findings extend the conventional picture of brush collapse and provide a basis for understanding the coupling between chain conformation and solvent microstructure in densely grafted polymer systems.

## 1. Introduction

Polymer brushes are systems composed of macromolecules chemically or physically attached at one end to a surface [1,2,3,4]. The properties of the chains grafted to the surface differ significantly from those of their counterparts in solution or in a dense polymeric liquid (free chains). In solution, depending on the temperature and quality of the solvent, the macromolecules change their conformation from a random coil to densely packed globules [5,6]. In the case of grafted chains, in addition to the quality of the solvent, the grafting density (the density of surface coverage by the grafted macromolecules) is also important. At least two regimes can be distinguished here. The first is the “mushroom” regime; at low grafting density, the chains are fairly separated from one another and do not overlap. The second regime, known as the “pancake” regime, occurs when the grafting density exceeds 1/(π$R_{g}^{2}$), where *R_g_* is the radius of gyration of a free, unperturbed chain (this is the case when the grafting points are, on average, separated by a distance of Rg). In this regime, due to the influence of beads belonging to neighboring chains, the chains expand perpendicular to the grafting surface, and this arrangement is called a polymer brush [7].

Due to their many functions and applications in biological, chemical, and technological processes, brushes have attracted considerable interest. Understanding the factors that influence the properties of brushes is therefore important for designing useful and intelligent polymer systems [1,8]. Brushes can be synthesized and studied using a variety of experimental techniques [1,7,9,10,11,12,13,14]. In actual experiments, polymer brushes can be obtained by attaching pre-polymerized chains to a surface (“grafting to”), by growing chains from initiators placed on the surface (“grafting from”), and through “grafting through,” which is, in a sense, a combination of these methods [14,15,16,17,18,19,20]. They have also been studied theoretically; however, due to their complexity, many questions remain unanswered [1,8,10]. The properties of the brushes were studied using simulations [9,21] employing molecular dynamics and dissipative particle dynamics [22,23,24,25,26,27,28,29,30,31], Monte Carlo [31,32,33,34,35,36,37,38,39,40,41,42,43,44,45,46,47], scaling theories, and theoretical considerations using the self-consistent field [31,48,49,50,51,52,53,54]. The simulations primarily examined scaling relationships regarding the sizes of polymers and brushes, as well as polymer density profiles, and found them to be close to the theoretically predicted values. The studies listed above primarily examine changes in the conformational behavior of polymer chains as the grafting density varies. The relationship between the structure of the chains and the brushes themselves and the quality of the solvent and temperature has been studied much less frequently [55,56]. Such relationships have already been determined experimentally [57,58,59,60], through theoretical analysis [53,54,61,62,63,64,65,66,67,68], and through computer simulations [30,69,70,71,72]. The latter have demonstrated scaling relationships between parameters describing the structure of the brushes and the chain length and grafting density. It was shown recently that the behavior of brushes built of long chains even at low grafting densities differs from that predicted by scaling theories; the scaling behavior of polymer density profiles was also different [73]. The structures formed at low temperatures were also examined [30,70]. Despite this extensive body of research on the structure of polymer brushes, no comprehensive analysis has yet been conducted covering a wide range of solvent quality, grafting density, and macromolecule length. The challenges stem from the complexity of macromolecular systems such as brushes, as most simulation algorithms fail in the case of weak solvents (low temperatures) and at high system densities. Furthermore, it should be emphasized that few studies published to date have accounted for the presence of explicit solvent molecules [30,70,73].

In this work, we present the results of studies on brush systems composed of flexible linear homopolymers grafted onto a flat and uniform surface. The surface was impermeable, and the polymers were of uniform length (monodisperse systems); their representation was coarse-grained, not taking into account atomic-level details, and based on a lattice. The system under study also contained explicit solvent molecules of the same size as the polymer beads. The main distinction of our approach is the simultaneous consideration of a broad range of grafting densities, chain lengths, and solvent quality. An explicitly represented solvent in a completely occupied system was another advantage of our model. The properties of this model, depending on the quality of the solvent, the grafting density, and the chain lengths, were determined using the Cooperative Motion Algorithm (CMA), a variant of the Monte Carlo method that allows for the study of high-density macromolecular systems. Consequently, systematic studies were conducted on the influence of these parameters on the structure of the brushes. Using the same model, the structure of individual free chains for different solvent qualities [74] and polymer brushes in a good solvent [73] was recently simulated using the same simulation method. This allows the properties of the systematically studied polymer brushes to be related to those of free chains and athermal brushes (in a good solvent).

## 2. Materials and Methods

The polymer brush under study consisted of flexible linear chains, one end of each of which was attached to the surface (the chains are flexible in the sense that no additional bending, angular, or torsional potential is imposed; it means that no additional energetic penalty for chain bending or torsion is included). A coarse-grained approximation was used, omitting details at the atomic scale, and each chain consisted of *N* identical beads. This is a coarse-grained representation in which the size of an element (a polymer bead, a solvent molecule) is on a scale much larger than the atomic scale. This size is determined by the distances between the lattice constant. Therefore, the model is intended to describe generic structural and conformational behavior rather than the exact molecular volume of a particular chemical species. The beads comprising the macromolecules as well as solvent molecules were placed in a face-centered cubic (fcc) lattice. This lattice was based on vectors of the form (±1, ±1, 0), (±1, 0, ±1), and (0, ±1, ±1) with a coordination number z = 12. The bonds connect the beads from adjacent nodes in the lattice; connected beads do not interact via potential from Equation (1). The surface was impenetrable, i.e., the plane and the portion of space on one side of it were excluded for objects in the Monte Carlo cell. No attractive or repulsive interactions were introduced between the surface and the polymer or solvent. The surface consisted of 2 × 10^4^ lattice units in directions parallel to the surface (the xy plane, 141 × 141). The chains were grafted onto the {1,1,1} plane, i.e., onto the plane of the closest packing in the fcc lattice, with periodic boundary conditions imposed exclusively in the x and y directions. Perpendicular to the grafting surface, the edge of the Monte Carlo cell was 100, and thus the system contained 10^6^ fcc lattice sites. In addition to polymer beads, the Monte Carlo cell also contained solvent molecules, whose positions were likewise restricted to the fcc lattice sites. All lattice sites in the cell were occupied by objects, that is, polymer beads or solvent molecules (incompressible lattice model).

The simple, single-parameter potential for interactions between objects *V_ij_* was defined as follows:

(1) $$V_{\mathrm{ij}}=\left\{\begin{array}{ll} 0 & \mathrm{for}\;r_{\mathrm{ij}}>l\\ E_{\mathrm{ab}}/k_{B}T & \mathrm{for}\;r_{\mathrm{ij}}=l\\ \infty & \mathrm{for}\;r_{\mathrm{ij}}<l \end{array}\right.$$

where *r_ij_* denotes the distance between a pair of objects, *l* is the lattice constant, *T* is the temperature, and *k*_B_ = 1 is the Boltzmann constant. Meanwhile, *a* and *b* denote the types of interacting objects: polymer (P) or solvent (S). Pairs of polymer beads connected by a bond are excluded from the contact energy calculations. An interaction scheme similar to that considered in the study on the collapse of free chains [74] was adopted; that is, the following interactions were assumed between polymer beads, solvent molecules, and between polymer beads and solvent molecules: *E_PP_* = *E_SS_* < 0 and *E_PS_* = 0. This corresponds to a situation in which the effective polymer–solvent incompatibility can be described by the Flory–Huggins parameter *χ*, defined in the standard way as

(2) $$\chi =\frac{z}{k_{B}T}\left[E_{\mathrm{PS}}-\frac{E_{\mathrm{PP}}+E_{\mathrm{SS}}}{2}\right]$$

where *z* is the lattice coordination number. The solvent quality is controlled by the Flory–Huggins parameter χ: the more positive the χ value is, the worse the solvent is.

A model constructed in this way allows for the simultaneous tracking of polymer chain conformations and the spatial arrangement of the solvent. An essential element of the model was therefore the presence of an explicit solvent, which allowed for the analysis of its local reorganization within the brush. In particular, it was possible to observe the effect of partial trapping of solvent molecules within the polymer layer structure, leading to the formation of heterogeneous domains rich in solvent and polymer. This effect, which is crucial for interpreting the results, is not present in effective solvent models.

The properties of the model brush were then simulated using the Monte Carlo method. Since the system was completely filled, the objects could not move over long distances, as all lattice sites in the system were occupied by other polymer beads and solvent molecules. Therefore, the Cooperative Motion Algorithm [75] was used. The basis of CMA is the idea of cooperative movement of system elements along identified closed paths in the lattice. Such paths are identified, and then the elements forming a given path are moved to an adjacent location in the lattice, provided that the integrity of the chains is maintained, bonds are not crossed, and the Metropolis condition is fulfilled. In a single Monte Carlo step, an attempt was made, on average, to move each object. The simulation procedure consisted of two stages. The first stage was the synthesis of the polymer chains forming the brush. The “grafting from” method was chosen because it allows for the synthesis of a brush with a high degree of grafting and for obtaining sufficiently equilibrated macromolecular systems for a high degree of grafting [15,16,36,37]. It corresponds to radical polymerization under conditions of controlled chain growth. The initiator was randomly placed on the surface at a given grafting density; the grafting density *σ* is defined as the ratio of the number of lattice sites occupied by the initiator to the total number of sites located on the grafting surface. Next, the entire Monte Carlo cell was filled with monomer, and a controlled, living, irreversible radical polymerization was carried out, assuming that the system was athermal—that is, without any interactions between the polymer and the monomer outside the excluded volume. (It is important to remember that the procedure simulating the polymerization process is based on a simplified coarse-grained model and does not represent the polymerization process at the atomic scale). The free end of a chain remains active until the polymer reaches a specified degree of polymerization (N beads). At that point, the polymerization step is complete for that macromolecule, while all other chains that have not reached that specified length continue to grow. During a given Monte Carlo step, an attempt is made to extend each chain by 1 bead. The first stage led to the formation of a system of an athermal polymer brush. After the polymerization reaction was stopped, the monomers that had not reacted were replaced with solvent molecules, and in the second stage, the system was equilibrated for fixed values of the parameters *N*, *σ*, and *χ* by performing between 10^6^ and 10^7^ Monte Carlo steps [76]. Equilibrium was determined based on the stabilization of the parameters defining the chain’s size, energy and polymer density profiles. Production Monte Carlo simulation runs were carried out. For a given set of parameters, each simulation consisted of 10^6^–10^7^ steps and was run 20–25 times, starting from different conformations, while for each independent simulation, a new random realization of the grafting pattern was generated to obtain adequate statistics. The parameters defining the brush structure were collected at appropriate time intervals to avoid correlation among them; these time intervals depended on *N*, *σ*, and *χ* (between 10^3^ and 10^5^ Monte Carlo steps). The statistical uncertainties of the estimated parameters (standard error of the mean from independent simulations) did not exceed 3%. The interaction parameter was gradually changed from χ/z 0.01 to 0.8 in order to avoid quenching.

The original data presented in the study are openly available in the Dane Badawcze UW repository (accessed 11 October 2026) at https://danebadawcze.uw.edu.pl/dataset.xhtml?persistentId=doi:10.58132/LPHC9O.

## 3. Results and Discussion

Simulations were performed for brushes composed of chains of equal length, each consisting of *N* = 20 to 120 beads. The simulations cover grafting densities, *σ* = 0.01 − 0.4, and the number of grafted chains were varied from 100 to 4000, depending on the selected grafting density. Its relation to the mean distance between grafting sites can be estimated from the surface-site density, and thus, the mean grafting-point separation covers values of ca. 1.6–ca. 10. The interaction potentials were set to *E_PP_* = *E_SS_* = −1 and *E_PS_* = 0, and the quality of the solvent was varied by changing the temperature. The brush was studied over a wide range of solvent quality, and the Flory–Huggins interaction parameter varied from good-solvent conditions (*χ*/*z* = 0.01) to poor-solvent conditions (*χ*/*z* = 0.8).

We will begin our description of the polymer layer’s structure by examining the behavior of the mean size of individual chains in the brush. The parameter used to describe the size is the mean square radius of gyration $\langle R_{g}^{2}\rangle$, defined as

(3) $$\langle R_{g}^{2}\rangle =\langle \frac{1}{N}\sum_{i=1}^{N}{\left(r_{i}-r_{\mathrm{cm}}\right)}^{2}\rangle$$

where ***r****_i_* denotes the coordinates of the i-th bead, and ***r****_cm_* denotes the coordinates of the center of mass.

**Figure 1:**
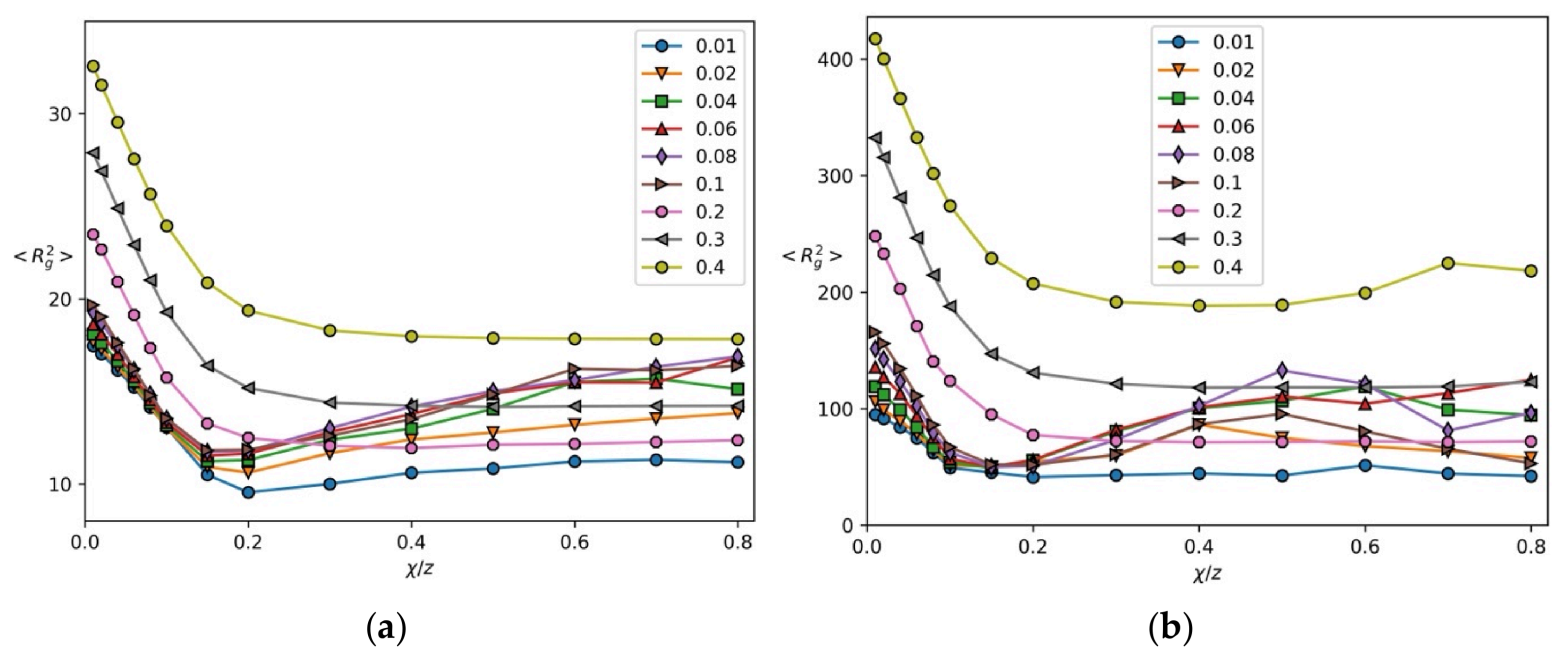
The mean square of the chain’s radius of gyration <$R_{g}^{2}$> vs. the parameter *χ*/*z*. The grafting densities *σ* are given in the inset. Cases for chains with *N* = 30 (**a**) and *N* = 120 (**b**).

Figure 1 shows the dependence of $\langle R_{g}^{2}\rangle$ on the parameter χ/z for various grafting densities *σ*. The distances are expressed in units of the lattice constant, energies are expressed in units of *k_B_*/*T*, the grafting density *σ* and the interaction parameter *χ*/z are dimensionless quantities (this applies to all figures). A clear difference can be seen in the behavior of chains at low (χ/z < 0.1) and high grafting densities. For short chains (Figure 1a shows changes in the sizes of chains with *N* = 30), at low σ, an increase in interaction strength initially (for χ/z < 0.2) leads to a significant decrease in chain sizes, followed by an increase, albeit at a slower rate. For high grafting densities across the entire range of interactions, chain sizes decrease, and this decrease is slower for χ/z > 0.2. For longer chains (Figure 1b, *N* = 120), the changes in chain size for higher grafting density remained unchanged. In contrast, for low grafting densities, we observe a temporary increase in size around *χ*/*z* = 0.5. These results can be compared to the case of spherical polymer brushes, where a local minimum in the size–temperature relationship was explained by the collapse of chains near the grafting site; at low densities, this led to the formation of isolated aggregates, while a further decrease in temperature caused longer chains to merge [77]. In any case, the curves shown in Figure 1a,b differ significantly from those for free chains studied within a similar model (coarse-grained representation, fcc lattice): in that case, the curves had distinctly classical S-shaped profiles [76].

**Figure 2:**
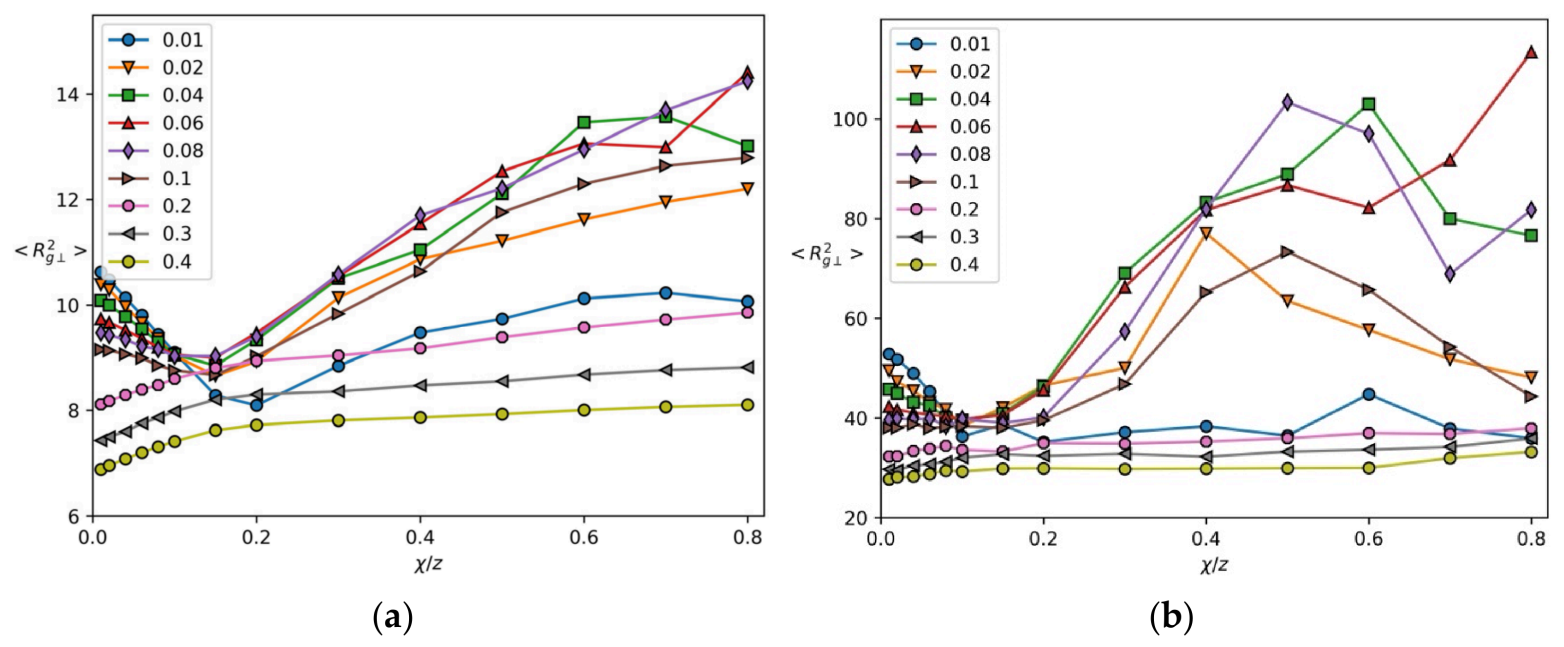
The component of the mean square radius of gyration of the chain <$R_{g}^{2}$_⏊_> perpendicular to the grafting surface vs. the parameter *χ*/*z*. Grafting densities *σ* are given in the inset. Cases for chains with *N* = 30 (**a**) and *N* = 120 (**b**).

The hypothesis presented above, which explains the non-monotonic behavior of the chain size, can be verified by analyzing the behavior of the component of the radius of gyration perpendicular to the grafting surface. Figure 2 shows the dependence of this parameter <$R_{g}^{2}$_⏊_> on the parameter *χ*/*z* for the same systems that were shown and discussed in Figure 1. For short chains (*N* = 30, Figure 2a), at low grafting densities, the behavior of <$R_{g}^{2}$_⏊_> is similar to $\langle R_{g}^{2}\rangle$, meaning that it decreases as the solvent quality decreases, but increases for *χ*/*z* < 0.2. In contrast, at high grafting densities, we observe a systematic decrease in chain size along this direction, and this decrease becomes more pronounced for a poor solvent (for *χ*/*z* < 0.2). Thus, for high values of *σ*, we observe a clear stretching of the chains in the direction perpendicular to the grafting surface. For long chains (*N* = 120, Figure 2b), the behavior of the component of the radius of gyration perpendicular to the grafting surface is more complex: a decrease in solvent quality initially leads to an increase in <$R_{g}^{2}$_⏊_>, after which this parameter behaves as it does for short chains. In both cases—for short and long chains—an increase or temporary rise in <$R_{g}^{2}$_⏊_> occurs mainly at the expense of a decrease in the value of <$R_{g}^{2}$_||_>. In summary, the changes in the value of this chain-size parameter differ from those obtained in Ref. [30], where they decrease monotonically, and the $R_{g}^{2}$_⏊_(*T*) curves were similar to those for free chains, i.e., they were S-shaped.

**Figure 3:**
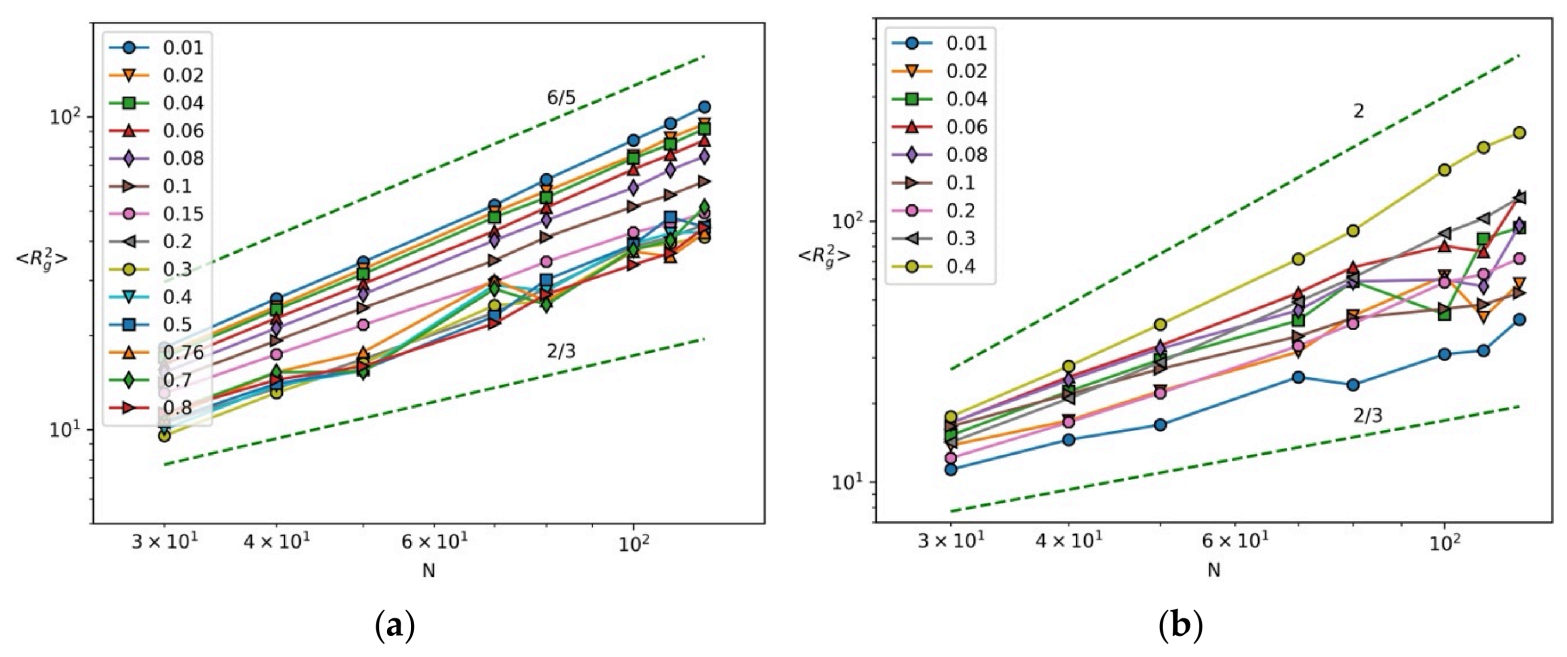
The mean square of the radius of gyration <$R_{g}^{2}$> vs. the chain length *N*. Cases for *σ* = 0.01 (**a**) and *χ*/*z* = 0.8 (**b**). The values of the parameter *χ*/*z* and the grafting density *σ* are given in the insets.

It is also worth examining how the chain size scales with the number of beads. Figure 3 shows the dependence of the mean square radius of gyration <$R_{g}^{2}$> on the number of beads in the chains, N. In each case shown, the expected relationship <$R_{g}^{2}$>~*N*^2ν^ holds, although significant size fluctuations are noticeable for poor solvents and higher grafting densities. Figure 3a shows the chain size at low grafting density (*σ* = 0.01) for several values of the parameter *χ*/*z*. It can be observed that the exponent 2ν varies from 6/5 (as predicted by theory for a good solvent) to 2/3 (as predicted by theory for a poor solvent), thus behaving similarly to free chains in a dilute solution, where we observe a transition from a “random coil” structure to a densely packed globule [76]. Similar exponents are predicted for systems in the mushroom regime by theories [56,57] and simulations using the dissipative particle dynamics method [25]. Figure 3b shows the effect of packing density on chain length in a weak solvent. As can be seen, at high densities, the exponent 2ν reaches a value of 2, which is characteristic of rigid rods. This value indicates that the so-called concentrated brush regime has been reached. An exponent close to 2 has already been reported in simulation results [25] and predicted by theoretical models [56,57].

**Figure 4:**
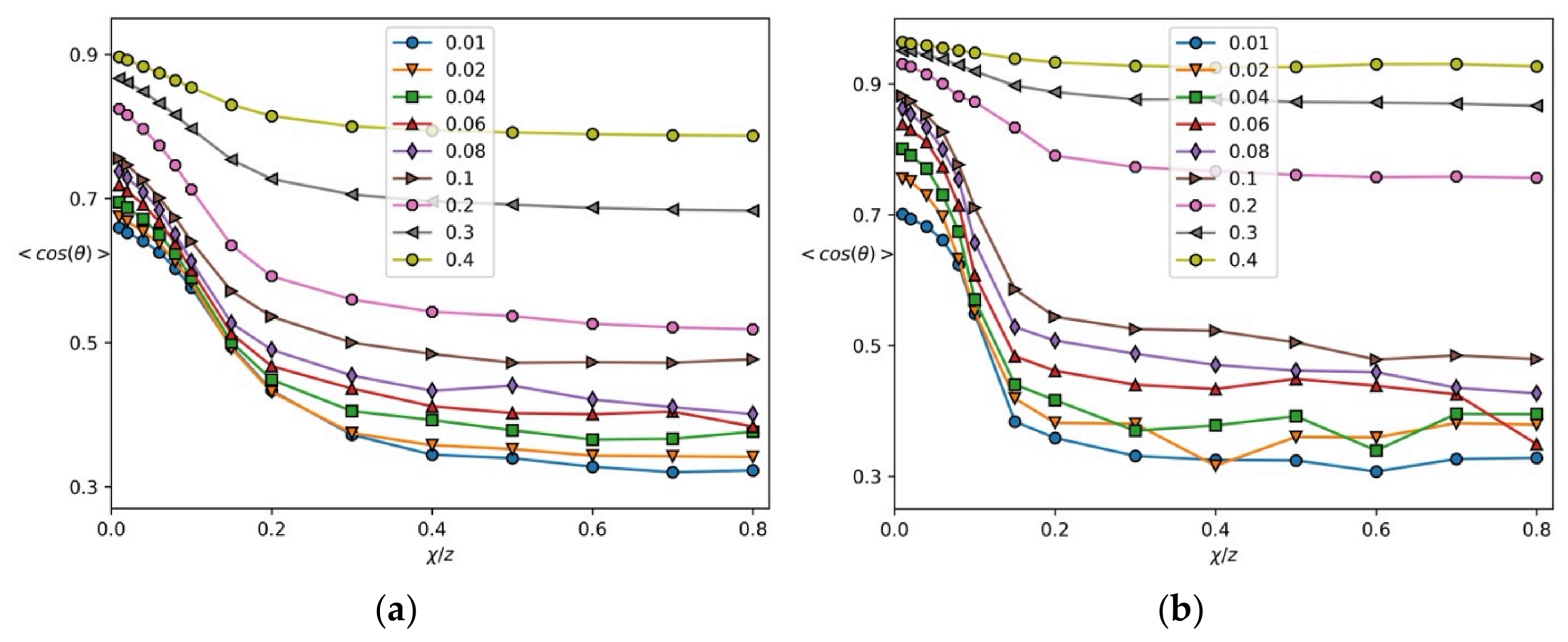
The average cosine of the angle between the vector connecting the grafting point and the free end of the chain vs. the parameter *χ*/*z*. Grafting densities *σ* are given in the inset. Cases for chains with *N* = 30 (**a**) and *N* = 120 (**b**).

The orientation of the chains in the brush can be determined by analyzing the average angle θ between the grafting plane and the vector connecting both ends of the chain. Figure 4 shows the relationship between the average cosine of this angle and the solvent quality *χ*/*z* for brushes composed of short and long chains. In general, it can be observed that, initially, as the value of *χ*/*z* increases, the value of <cos(*θ*)> decreases rapidly, and only when *χ*/*z* > 0.2 does the decrease become more gradual, especially for low grafting densities *σ*. The values of the angle θ, of course, also depend on the grafting density and, as expected, increase with increasing grafting density, reaching values corresponding to a right angle. Once again, a quantitative difference in the behavior of the chains is evident between low and high grafting densities: the angle *θ* for the latter is significantly larger across the entire temperature range studied; these differences increase with increasing chain length. In summary, grafting density has a strong influence on chain orientation, especially under poor-solvent conditions. A worsening in solvent quality leads to reorientation only for short chains and, for long chains, only at low grafting densities.

**Figure 5:**
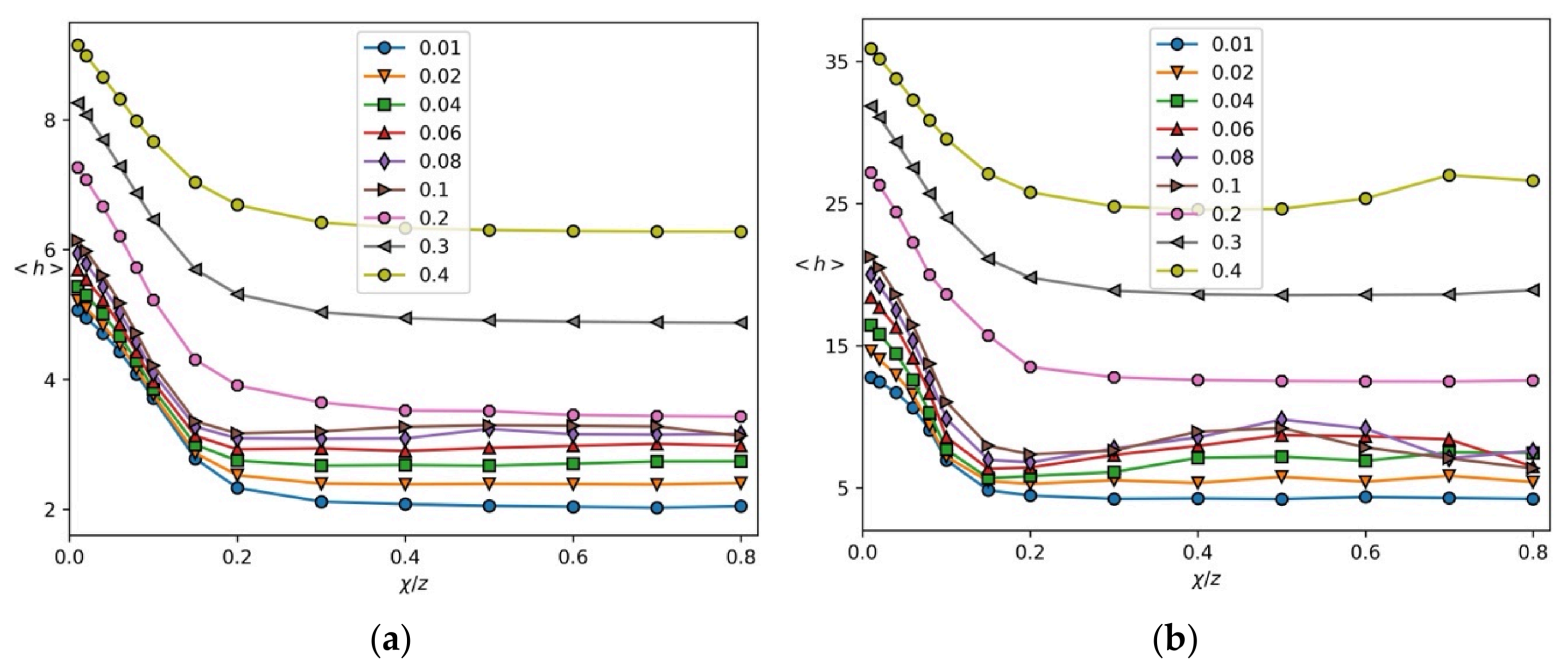
The average brush height <*h*> vs. the parameter *χ*/*z*. Grafting densities *σ* are given in the inset. Cases for chains with *N* = 30 (**a**) and *N* = 120 (**b**).

Further analysis of the brush structure was conducted based on its macroscopic geometric properties and density profiles. One such macroscopic parameter is the average height of the brush <*h*>, defined as the first moment of the density profile:

(4) $$<h>=\frac{\sum_{z=1}^{z_{\max}}\varphi \left(z\right)z}{\sum_{z=1}^{z_{\max}}\varphi \left(z\right)}\;$$

where *φ*(*z*) is the polymer density along a direction perpendicular to the grafting surface. Figure 5 shows the dependence of the average height <*h*> on the solvent quality parameter for brushes composed of short and long chains. As in the case of the radius of gyration, we can distinguish two regimes of brush height behavior: for *χ*/*z* < 0.2, as the solvent quality deteriorates, the height of the polymer layer decreases significantly and then stabilizes in the region of poor solvent quality. For intermediate grafting densities (*σ* between 0.06 and 0.1), a slight maximum emerges on the *h*(*χ*/*z*) curves near *χ*/*z* = 0.5. Thus, a transition between the good-solvent and poor-solvent regimes, typical of polymer systems, can be observed; however, there is no clearly developed universal power-law scale as a function of grafting density. In particular, only a weak agreement with the classical predictions of scaling theory [54] is observed, suggesting that the presence of an explicit solvent and lattice constraints significantly modify the entropic–energetic equilibrium of the system. The non-monotonic behavior observed in poor-solvent conditions (*χ*/*z* ≥ 0.3) can also be attributed to a crossover between single-chain collapse and solvent trapping-induced re-swelling at higher effective confinement (see below).

**Figure 6:**
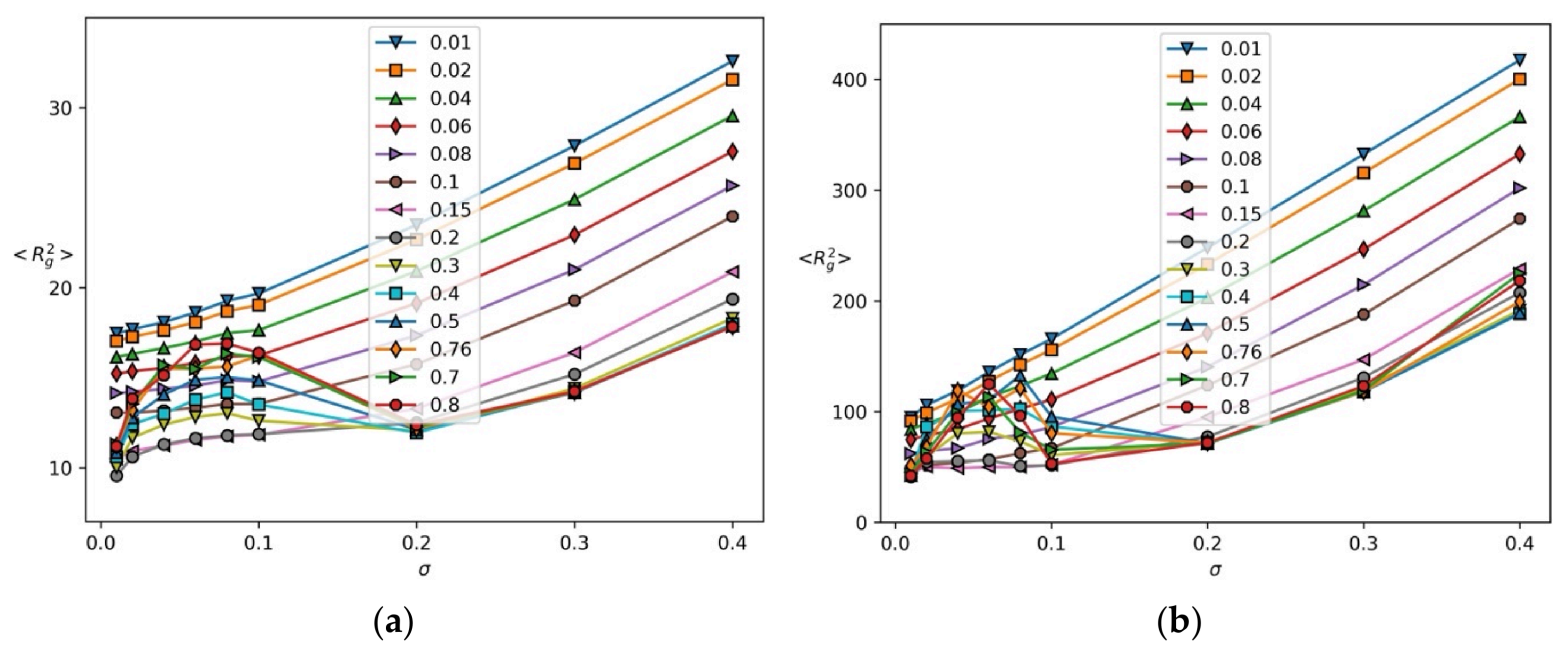
The mean square radius of gyration <$R_{g}^{2}$> vs. the grafting density *σ* for different solvent qualities *χ*/*z*. Cases for chains with *N* = 30 (**a**) and *N* = 120 (**b**). The values of the parameter *χ*/*z* are given in the inset.

**Figure 7:**
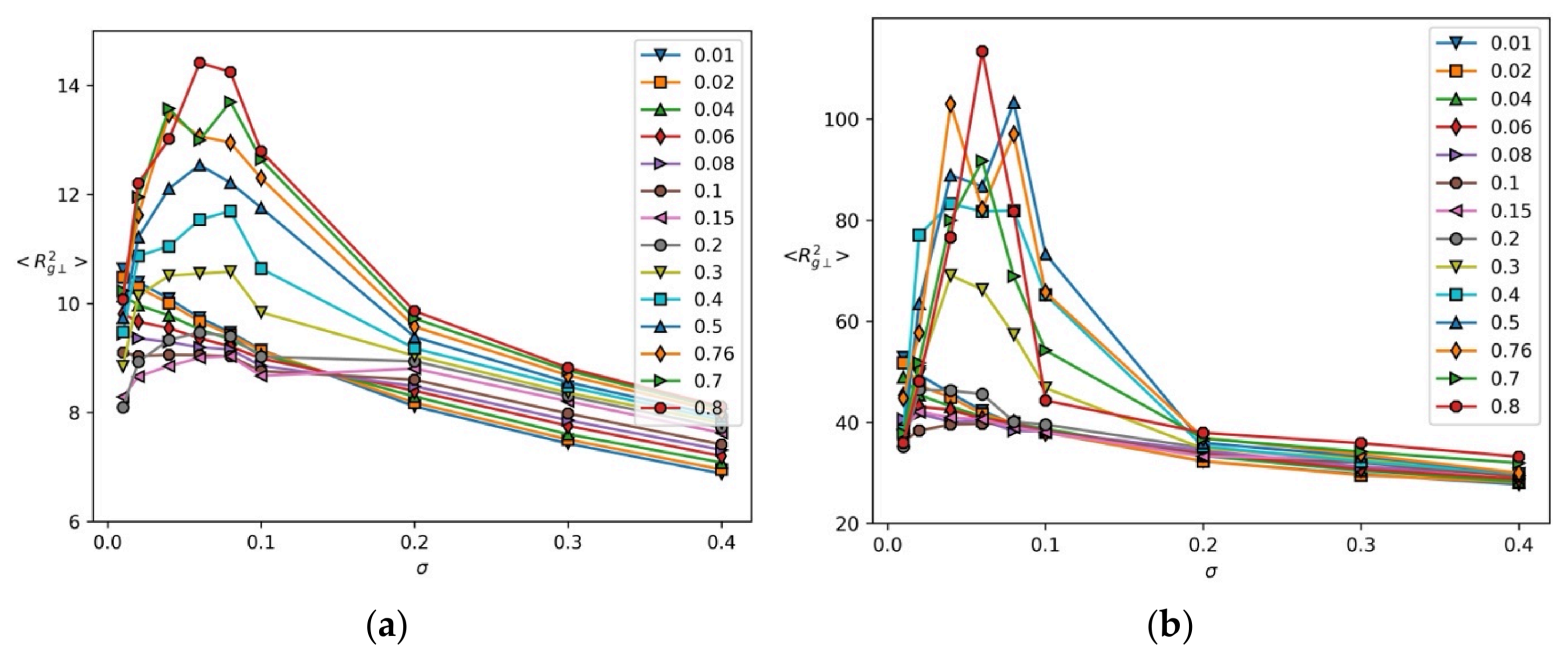
The component of the mean square radius of gyration of the chain perpendicular to the grafting surface <$R_{g}^{2}$_⏊_> vs. the grafting density *σ*. Cases for chains with *N* = 30 (**a**) and *N* = 120 (**b**). The values of the parameter *χ*/*z* are given in the inset.

A more complex picture emerges when analyzing the relationship between the parameters describing the brush and the grafting density. Figure 6 shows the effect of *σ* on chain sizes, i.e., on the value of the chain’s mean square radius of gyration <$R_{g}^{2}$>, and Figure 7 shows the component of the radius of gyration perpendicular to the grafting surface <$R_{g}^{2}$_⏊_>. For good and moderately poor solvents, an increase in grafting density leads to a gradual increase in chain size, which can be attributed to the strengthening of the excluded volume effect. In contrast, under poor solvation conditions (χ/z ≥ 0.3), a distinct non-monotonicity emerges; both <$R_{g}^{2}$> and <$R_{g}^{2}$_⏊_> exhibit a maximum at low grafting densities (*σ* < 0.2), and the lower the solvent quality, the greater the maximum on the <$R_{g}^{2}$_⏊_>(*σ*) curve. This means that, in this range of parameters, the system does not behave like a homogeneous elastic layer but exists in a transition state in which local chain collapse coexists with their partial re-expansion, forced by geometric constraints and local changes in solvent density. The re-expansion at intermediate σ reflects solvent accumulation inside the brush.

**Figure 8:**
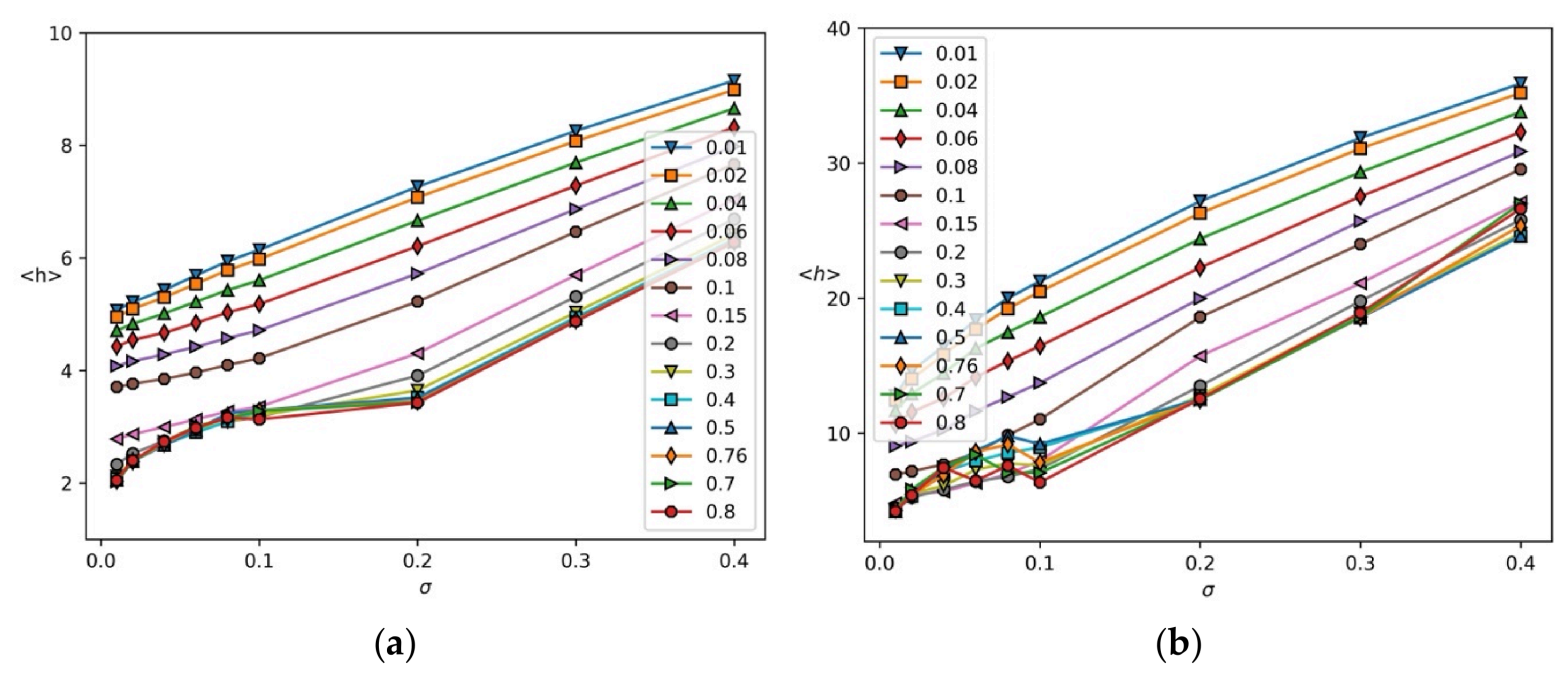
The average brush height <*h*> vs. the grafting density *σ*. Cases with *N* = 30 (**a**) and *N* = 120 (**b**) chains. The values of the parameter *χ*/*z* are given in the inset.

The relationship between the average brush height and grafting density is shown in Figure 8. Across the entire range of grafting densities, the brush height increases with *σ*. In general, it is neither a constant value nor a power-law increase, although theories suggest that *h*~*σ*^0^ for the mushroom regime and *h*~*σ*^1/3^ for the pancake regime [78], which has been confirmed by experimental studies [13,56]. We observe, however, that for short chains, the increase in layer height is slower at low grafting densities, and for a poor solvent, it stabilizes between *σ* = 0.1 and 0.2, closely approximating the behavior *h*~*σ*^0^. For long chains, the brush height also increases with grafting density. Only under conditions of a moderately poor solvent (*χ*/*z* < 0.2) and low grafting densities do we observe non-monotonic changes in brush height. This non-monotonic behavior corresponds to changes in chain length (Figure 6 and Figure 7). The relationships between brush height and grafting density for a moderately poor solvent are generally similar to those for a good solvent [73]. Experimental results for brushes made of poly(methyl methacrylate) in acetone and methanol, however, show that this exponent for a good solvent varies from 0.33 to 0.60 during the transition from the mushroom regime to the brush regime, whereas in a poor solvent, it is 0.80 over a wide range of grafting densities [57]. Deviations from monotonic scaling in poor-solvent conditions can be associated with solvent redistribution within the brush.

**Figure 9:**
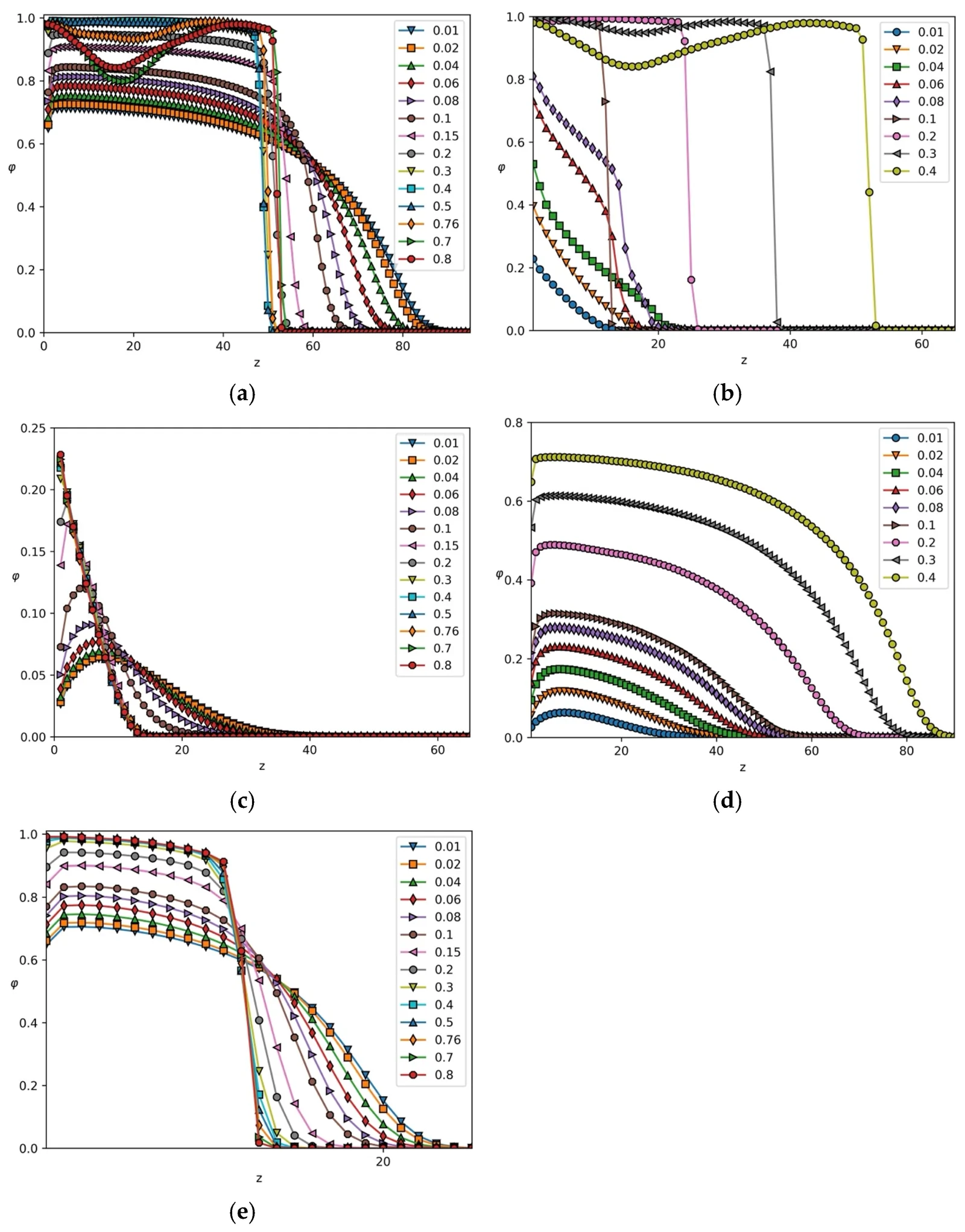
Polymer density profiles in the brush. Chain *N* = 120, grafting density *σ* = 0.4 (**a**), chain *N* = 120, parameter *χ*/*z* = 0.8 (**b**), chain *N* = 120, grafting density *σ* = 0.01 (**c**), chain length *N* = 120, parameter *χ*/*z* = 0.01 (**d**), chain length *N* = 30, grafting density *σ* = 0.4 (**e**). The values of the parameter *χ*/*z* and the grafting density *σ* are given in the insets, respectively.

Figure 9 shows the density profiles for a brush consisting of chains of length *N* = 120 with different grafting densities and solvent qualities. It can be observed that, for brushes characterized by a high *σ*, there is a very sharp interface between the polymer and the solvent. This interface shifts with the quality of the solvent (Figure 9a). Similar density profiles were obtained in molecular dynamics simulations of a coarse-grained brush model, although this system was only half-filled with polymer and solvent [30], as well as in atomistic simulations of brushes made of poly(ethylene oxide) [27]. Going back to our results, in a poor solvent, only a high grafting density yields similar density distributions (Figure 9b). In contrast, for brushes with low grafting density, the profiles look completely different—there are no regions of high and constant polymer density, and the polymer concentration decreases rapidly with increasing distance from the grafting surface. For a good solvent (Figure 9d), we observe the shape of the distributions reported in other studies [24]. The change in the shape of the distributions when going from a good to a poor solvent is similar to those obtained for other continuous coarse-grained models [24]. Inside brushes with high grafting density and in a poor solvent, we observe density minima in the polymer layer; a similar, though much less pronounced, phenomenon has already been observed [24]. We are thus dealing with the presence of a certain amount of solvent near the midpoint of the polymer layer’s height. This effect appears after the chains collapse but then weakens as the quality of the solvent continues to weaken. The presence of solvent in the polymer layer is also influenced by the grafting density: it appears once the mushroom regime is reached and intensifies as the grafting density increases. Experimental studies have observed a similar effect: for high grafting densities, the amount of solvent in the brush increased with *σ*, but only in a good solvent. This phenomenon was explained by the non-uniformity of brush densities, which allows them to absorb solvent near the surface [57]. It becomes pronounced for sufficiently long chains within the range investigated. Figure 9e shows the polymer density profiles for a chain of *N* = 30 and a grafting density of *σ* = 0.4. It can be seen that the polymer concentration is no longer close to unity over almost the entire range of brush height but decreases slightly with distance from the grafting plane. There is also clearly no solvent inside the brush. The shape of the density profiles for short chains is close to those predicted theoretically [66]. In summary, poor-solvent conditions correspond to an increase in the effective preference for polymer–polymer and solvent–solvent contacts relative to polymer–solvent contacts. Consequently, the chains tend to collapse locally and form polymer-rich regions. It should be emphasized that, even after the system has reached equilibrium, there is still solvent present in the brush. It is important to remember that the solvent also interacts with itself, tending to form aggregates. The complete removal of the solvent from the polymer brush is restricted by the geometry of the densely grafted system. For a grafting density of 0.4, the solvent content in the brush ranges from 3% (*χ*/*z* = 0.6) to 8% (χ/z = 0.8). Thus, solvent trapping in the present model emerges from the competition between unfavorable polymer–solvent interactions, polymer chain collapse, chain packing, and the geometrical and incompressibility constraints of the fully occupied lattice. However, it is important to remember that the system we studied, although large, was nevertheless finite.

The key to understanding the apparent inconsistency between Figure 6 and Figure 7 (peaks for low σ values) and Figure 9 (the strongest anomalies for high *σ* values) lies in analyzing the structure of the solvent in the brush. The density profiles (Figure 9) show that, under conditions of high grafting density and a poor solvent, a non-uniform structure forms, in which the solvent is not merely a background phase but creates locally enriched regions within the brush. This leads to the formation of regions with reduced polymer density and a non-uniform distribution of the material in the direction normal to the surface. From a thermodynamic perspective, this phenomenon can be interpreted within the Flory–Huggins framework as the result of competition between the enthalpic contribution of the parameter *χ*, which promotes polymer–solvent demixing, and the mixing entropy of the solvent, which favors its presence in the layer. In the classical mean-field approach, this equilibrium leads to a homogeneous response of the system. However, in the case under consideration, additional geometric constraints and the absence of free sites in the lattice prevent the solvent from being fully displaced, leading to its trapping within the brush. The observed solvent redistribution is consistent with a confinement-induced solvent contribution that can oppose or modify the tendency of the polymer chains to collapse. As a result, the brush undergoes spontaneous microstructural separation, in which polymer-rich and polymer-poor regions coexist. It is precisely this mechanism that accounts for the strong anomalies in the density profiles (Figure 9), which do not necessarily translate directly into global conformational measures (Figure 6 and Figure 7), since they primarily concern spatial reorganization rather than total compression of the system. A summary of the results therefore indicates that a polymer brush in a poor solvent exhibits two qualitatively different response regimes: (i) a low-grafting-density regime, in which non-monotonic conformational changes in individual chains dominate, resulting from the competition between the Flory–Huggins energy and chain entropy, and (ii) a high-grafting-density regime, in which collective reorganization of the solvent plays a key role, leading to the formation of a non-uniform structure.

**Figure 10:**
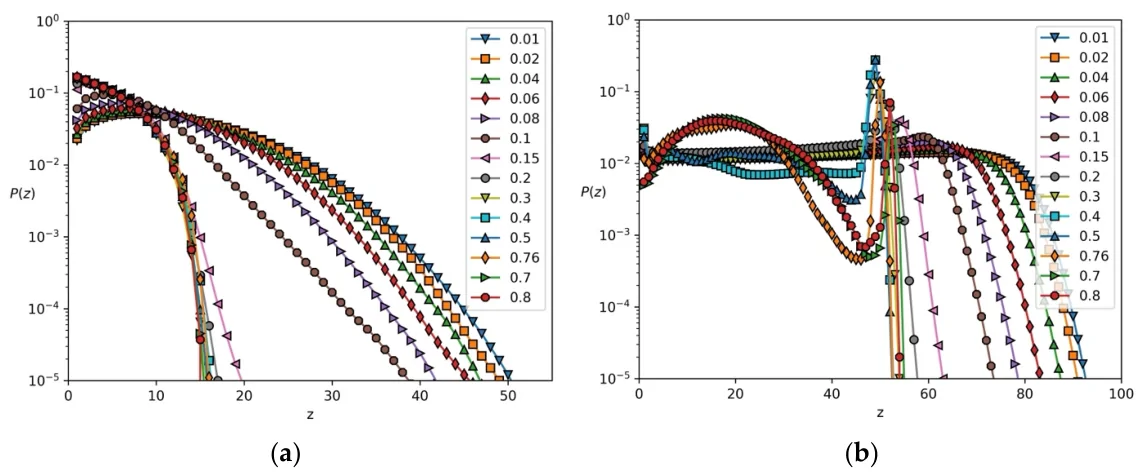
The normalized polymer–solvent density-overlap function *P*(*z*) for a chain with *N* = 120. The case of grafting density *σ* = 0.01 (**a**) and grafting density *σ* = 0.4 (**b**). The values of the parameter *χ*/*z* are given in the insets.

From the results presented above, we know that solvent penetration into the brush varies with morphological changes in the system caused by grafting density, chain length, and solvent quality. The degree of solvent penetration into the brush can be described using the normalized polymer–solvent density-overlap function at a given distance from the grafting surface, defined as

(5) $$P\left(z\right)=\frac{\varphi_{p}(z)\cdot \varphi_{s}(z)}{\sum_{z=1}^{z_{\max}}\varphi_{p}(z)\cdot \varphi_{s}(z)}\;$$

where *φ*_p_(*z*) and *φ*_s_(*z*) correspond to the concentrations of the solvent and the polymer, respectively, for a given coordinate z. Figure 10 shows the probability of contact between the polymer and the solvent for a given brush layer *z*. For low grafting densities, i.e., the mushroom regime (*σ* = 0.01), maxima are clearly visible on the curves, indicating that the probability of solvent molecules being present is higher near the grafting surface than at the center of the layer. The maximum on the *P*(*z*) curves shifts slightly away from the grafting surface as the solvent quality deteriorates (Figure 10a). Further worsening of solvent quality (*χ*/*z* > 0.1) no longer has a significant effect on the presence of solvent in the brush; the maxima on the *P*(*z*) curves also disappear. Increasing the grafting density (*σ* = 0.4) and reaching the brush regime drastically changes the picture, as shown in Figure 10b. Worsening of the solvent quality up to *χ*/*z* > 0.15 does not significantly affect the value of the probability *P*(*z*); it is nearly constant and independent of *χ*/*z*; a slight increase should be noted throughout the brush region. Further deterioration of the solvent quality leads to interesting behavior of the probability. In addition to a maximum at one-third of the polymer layer thickness, there is also a significant decrease in the number of polymer–solvent interactions at distances not too far from the brush surface.

## 4. Conclusions

In this paper, we systematically studied the structure of polymer brushes (monodisperse homopolymers) using a simplified, coarse-grained model. It should be emphasized that the model system was completely filled with polymer and solvent. The properties of this model were determined using the Cooperative Motion Algorithm, a variant of the Monte Carlo method that is effective in dense soft-matter systems, which allowed us to simulate, over a wide range, the effects of chain length, grafting density, and solvent quality on the structure of the brushes. The present model is coarse-grained and does not describe a specific polymer chemistry, solvent, or surface chemistry. Therefore, the values of N, σ and χ should not be interpreted as direct quantitative predictions for a particular experimental material without additional calibration. Nevertheless, the model provides experimentally testable qualitative predictions. In particular, the simulations predict that under poor-solvent conditions and sufficiently high grafting density, solvent redistribution can produce non-uniform solvent-enriched regions within the polymer brush.

It was demonstrated that both grafting density and solvent quality strongly influence the conformation of individual chains and the macroscopic structure of the entire polymer brush. For high grafting densities, typical polymer brush behavior was observed, in which interactions between neighboring chains lead to their stretching perpendicular to the surface, while, on the other hand, a worsening in solvent quality caused the brush to gradually collapse, reducing its height. In contrast, for low grafting densities, non-monotonic changes in the radius of gyration and its component perpendicular to the surface were observed, particularly for long chains. This result indicates the presence of more complex structural processes associated with the local collapse of macromolecules and the potential for aggregate formation. Further analysis revealed that, under conditions of high grafting density and poor solvent, a heterogeneous structure forms in the polymer brush, including areas enriched with solvent. This effect suggests that the solvent does not merely serve as a homogeneous background medium but becomes an active structural component of the brush. The proposed interpretation within the Flory–Huggins theory framework suggests that the observed behavior results from competition between the enthalpic contribution of the parameter *χ*, which promotes chain collapse, the mixing entropy of the solvent, and chain packing. These results emphasize that a description of polymer brushes under poor solvation conditions must account for the coupling between chain conformation and the microstructural distribution of the solvent. This behavior differs from the classical predictions of mean-field theory and constitutes a significant extension of the description of polymer brushes under poor solvation conditions.

## Data Availability

The original contributions presented in this study are included in the article. Further inquiries can be directed to the corresponding author.
